# Rebalancing polyamine levels to treat Snyder–Robinson syndrome

**DOI:** 10.15252/emmm.202318506

**Published:** 2023-09-15

**Authors:** Susan K Gilmour

**Affiliations:** ^1^ Lankenau Institute for Medical Research Wynnewood PA USA

**Keywords:** Genetics, Gene Therapy & Genetic Disease, Metabolism

## Abstract

Snyder–Robinson syndrome (SRS) is a rare genetic disorder characterized by intellectual disability and delayed development beginning early in childhood. It was first described in a single family in 1969 as a sex‐linked disorder (Snyder & Robinson, 1969) and has since been only identified in less than 100 individuals worldwide. Inherited in an X‐linked recessive pattern, SRS has only been identified in males thus far. Snyder–Robinson syndrome primarily affects the nervous system and skeletal tissues and is caused by loss‐of‐function mutations in the gene encoding spermine synthase (SMS), a polyamine biosynthesis enzyme. Affected males display a collection of clinical features including intellectual disability ranging from mild to profound, speech and vision impairment, osteoporosis, hypotonia, and increasing loss of muscle tissue with age, kyphoscoliosis, seizures, and distinctive facial features including a prominent lower lip and facial asymmetry. Currently, there is no cure or treatment for this debilitating disorder aside from symptom management.

## Graphical abstract

*S. Gilmour* discusses the study by Stewart *et al*, in this issue of *EMBO Mol Med*, that proposes the use of 2‐difluoromethylornithine (DFMO) to rebalance the relative levels of the polyamines spermidine and spermine in order to treat Snyder–Robinson syndrome patients.

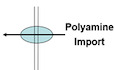

Snyder–Robinson syndrome results from an inactivating mutation in the spermine synthase (*Sms*) gene that is located on the X chromosome at Xp21.3‐p22.12 (Lauren Cason *et al*, 2003). Spermine synthase catalyzes the biosynthesis of the polyamine spermine by transferring the aminopropyl group of decarboxylated S‐adenosylmethionine (dcSAM) onto its precursor polyamine, spermidine. The SMS deficiency in SRS causes a decrease in the amount of spermine due to a partial dysfunction of SMS and an accumulation of spermidine resulting in an increased spermidine/spermine ratio. This ratio tends to be higher with more severe symptoms of SRS.

Polyamines (spermidine, spermine, and their precursor putrescine) are small polycations that are essential for all cell growth, differentiation, and development (Pegg, 2016). Polyamines interact with negatively charged macromolecules including nucleic acids, chromatin, ion channels, proteins, and phospholipids, thus affecting a wide variety of biological pathways. Alterations in polyamine levels have been associated with aging and a variety of diseases including cancer, inflammation, and neurological disorders (Sagar *et al*, 2021; Holbert *et al*, 2022). Not surprisingly, polyamine metabolism must be tightly regulated through biosynthesis, catabolism, uptake, and excretion in order to maintain normal cellular physiology. Spermine synthase deficiency is also associated with excessive spermidine catabolism that generates reactive oxygen species leading to lysosomal defects and mitochondrial dysfunction that have been shown to play a critical role in neurodegenerative diseases (Li *et al*, 2017). Although cells derived from SRS patients are capable of importing exogenous spermine, administration of spermine by intraperitoneal injection or via dietary supplementation has been unsuccessful in treating SRS patients (Wang *et al*, 2009).

In this issue of *EMBO Molecular Medicine*, Stewart *et al* (2023) describe the potential use of 2‐difluoromethylornithine (DFMO) to rebalance the relative levels of the polyamines spermidine and spermine to treat SRS patients. 2‐Difluoromethylornithine is an FDA‐approved inhibitor of polyamine biosynthesis that is currently used to treat parasitic infections and hirsutism. Recognizing that most SRS patients have hypomorphic mutations of the *Sms* gene causing reduced SMS activity and reduced spermine levels, the authors hypothesized that DFMO may rebalance spermidine/spermine ratios since polyamine metabolism is highly regulated by a series of compensatory feedback mechanisms. Indeed, DFMO inhibition of ornithine decarboxylase results in an expected decrease in putrescine and spermidine in SRS cells but also an increase in the production of dcSAM (Fig 1). This increase in dcSAM levels boosts spermine formation only in SRS cells that retain some SMS activity. In addition, DFMO has been shown to increase polyamine import into cells from extracellular dietary or intestinal bacterial flora sources. Using a novel methylated spermine mimetic that is resistant to catabolism by amine oxidases, they found that DFMO increases the uptake of spermine or the spermine mimetic in SRS cells, thus rebalancing the relative intracellular levels of spermidine and spermine and restoring cell growth to wild‐type levels.

**Figure 1:**
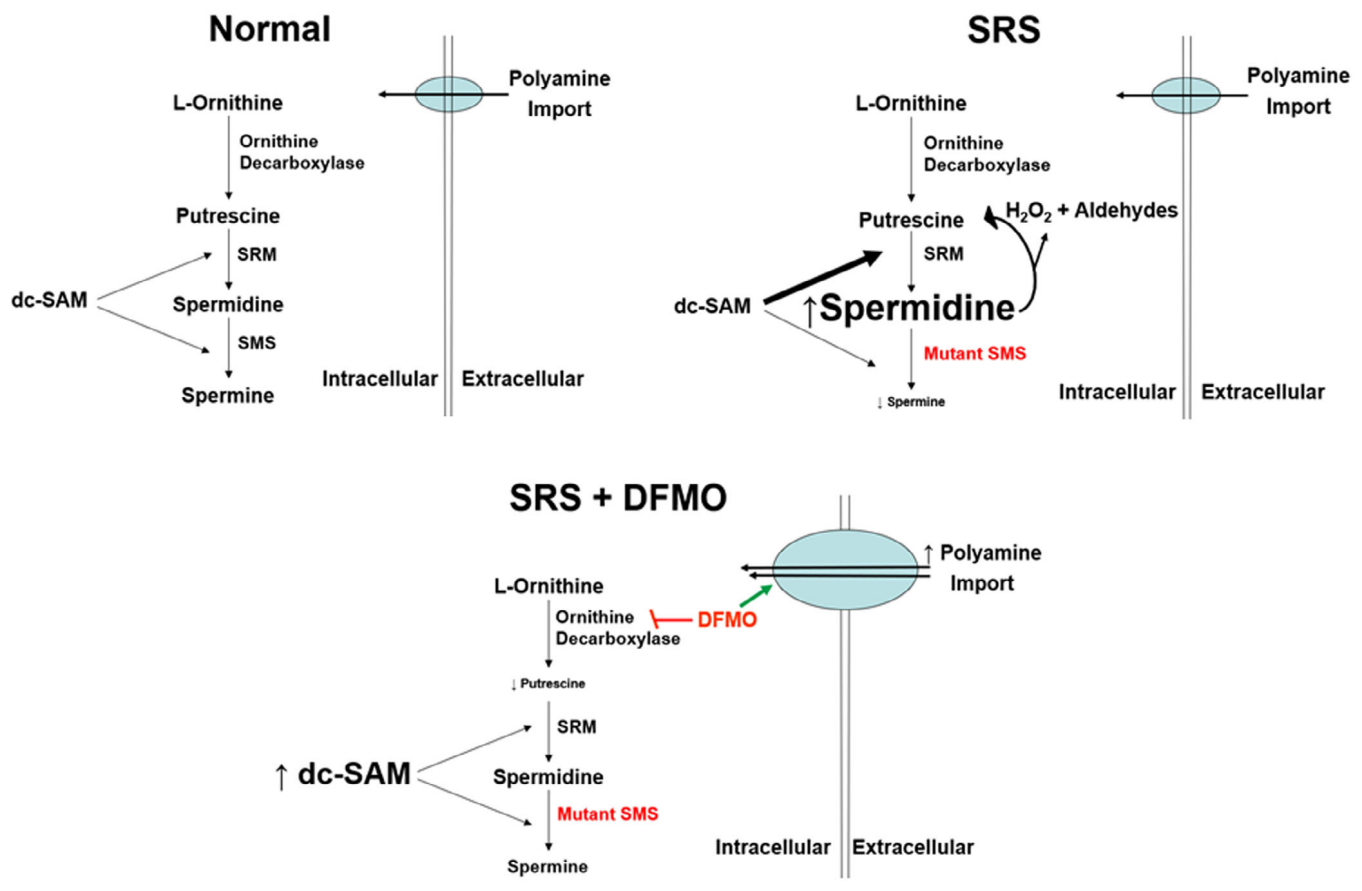
Diagram of changes in polyamine metabolism caused by SMS deficiency in SRS and normalization of spermidine/spermine ratios following DFMO treatment Both spermidine synthase (SRM) and SMS are aminopropyltransferases for catalyzing the formation of spermidine and spermine, respectively. Hypomorphic *Sms* mutations lead to spermidine buildup followed by increased spermidine catabolism and accumulated catabolic metabolites, H_2_O_2_ and aldehydes. 2‐Difluoromethylornithine rebalances the spermidine/spermine ratio in SRS cells with diminished loss of SMS activity by increasing production of dcSAM to facilitate spermine synthesis and by increasing cellular import of polyamines.

The search for a pharmacologic treatment for SRS has been hampered by limited *in vivo* models. Hemizygous *Gy* male mice have an X chromosomal deletion that inactivates the *Sms* gene and the downstream gene phosphate regulating endopeptidase homolog, X‐linked (*PHEX*), making it difficult to separate the effects of SMS deficiency and loss of PHEX function (Wang *et al*, 2009). Previous studies with *Gy* mice revealed that these mice displayed a rapid and lethal toxicity to DFMO (Wang *et al*, 2009). However, the complete loss of SMS activity in *Gy* mice does not mimic the diminished loss of SMS activity in SRS patients who typically have hypomorphic *Sms* mutations. A Drosophila model with the *dSms* gene knocked out (Li *et al*, 2017) was used to demonstrate that DFMO increases the life span of these flies when using medium containing spermine. Despite their lack of SMS activity, DFMO treatment rescued the *dSms* ^*−/−*^ flies presumably by increasing cellular uptake of the exogenous spermine from the fly medium to increase their life span, thus providing evidence of a possible therapeutic benefit using DFMO. Future preclinical studies using a newly generated mouse model of SRS that harbors the clinically relevant *Sms* ^*G56S*^ mutation will provide much needed data to evaluate to what extent DFMO and/or the methylated spermine mimetic can rescue SRS patient‐relevant phenotypes that remain to be identified in this mouse model. In addition, SRS primarily affects the nervous system and skeletal tissues, suggesting that different cell types have varying tolerances to SMS deficiency and polyamine imbalances. Many studies focused on SRS, including this paper, have used fibroblast and lymphoblastoid cells cultured from SRS patient tissues. Thus, future studies using more phenotypically relevant SRS cell types are needed to explore their differential sensitivities to possible treatments such as DFMO and spermine prodrugs (Tantak *et al*, 2021). Since additional polyamine genetic disorders have recently been identified that also display neurological defects (Prokop *et al*, 2021), finding pharmacological treatments that can correct polyamine imbalances in relevant cell types will be helpful for treating not only SRS but also other polyamine‐associated neurological disorders. Overall, this study (Stewart *et al*, 2023) has highlighted the importance of rebalancing the relative levels of the polyamines spermidine and spermine to more normal ratios by pharmacologically exploiting inherent cellular control mechanisms in polyamine metabolism. Using this mechanistic approach, DFMO offers hope as a therapeutic approach to alleviate the symptoms of SRS. A better understanding of how different *Sms* mutations disturb the spermidine/spermine ratio to cause varying degrees of SRS phenotypes in different tissues will ultimately lead to successful and safe treatments for this polyamine disorder.
